# Higher Temperatures, Higher Solar Radiation, and Less Humidity Is Associated With Poor Clinical and Laboratory Outcomes in COVID-19 Patients

**DOI:** 10.3389/fpubh.2021.618828

**Published:** 2021-03-19

**Authors:** Mahmood Yaseen Hachim, Ibrahim Y. Hachim, Kashif Naeem, Haifa Hannawi, Issa Al Salmi, Suad Hannawi

**Affiliations:** ^1^Mohammed Bin Rashid University of Medicine and Health Sciences, Dubai, United Arab Emirates; ^2^College of Medicine, University of Sharjah, Sharjah, United Arab Emirates; ^3^Ministry of Health and Prevention (United Arab Emirates), Dubai, United Arab Emirates; ^4^The Royal Hospital, Muscat, Oman

**Keywords:** COVID-19, SARS–CoV−2, environmental, weather, pandemic (COVID-19)

## Abstract

**Background:** The COVID-19 pandemic varies between countries, with suggestions that weather might contribute to the transmission mode, disease presentation, severity, and clinical outcomes. Yet the exact link between climate and COVID-19 is still not well-explored.

**Objectives:** This study aimed to evaluate the effect of hot geographical region weather [like United Arab Emirates (UAE)] on COVID-19 clinical profile and outcomes. Temperature, wind speed, cloud cover, precipitation, and other weather-related variables were studied concerning COVID-19 patients outcomes and laboratory results.

**Methodology:** A total of 434 COVID-19 positive patients admitted between January and June 2020, were recruited from Al Kuwait Hospital, Dubai, UAE. Temperature, wind speed, cloud cover, and precipitation rate were retrieved from history+ for the day when COVID-19 patients presented to the hospital. These weather parameters were correlated with COVID-19 clinical and laboratory parameters.

**Results:** Our results showed that patients needed admission in days with higher temperatures, higher solar radiation, and less humidity were associated with higher deaths. This association can be linked to the association of these weather parameters with age at diagnosis; higher C-reactive protein (CRP), neutrophil count, white cell count (WCC), aspartate aminotransferase (AST), and alkaline phosphatase (ALP); and lower lymphocyte count, estimated glomerular filtration rate (eGFR), hemoglobin (Hb), Na, and albumin, all of which are considered poor prognostic factors for COVID-19.

**Conclusion:** Our study highlighted the importance of weather-related variables on the dynamics of mortality and clinical outcomes of COVID-19. The hot weather might makes some people, especially those with comorbidities or older ages, develop aggressive inflammation that ends up with complications and mortality.

## Introduction

The exact link between weather and COVID-19 spread is still not well-explored, although a few reports claimed that warm weather can slow down such spread and can help in predicting which geographic areas in different countries can have a higher risk of spread (1). One of the COVID-19 pandemic characteristics is a very rapid spread and high mortality rates in countries north of the equator known to have low seasonal air temperatures (2). Such countries with low humidity are suspected to favor the transmission and survival of SARS-COV-2 (3). Such a link is not surprising for the virus family as Middle East respiratory syndrome coronavirus (MERS-CoV) human cases in Saudi Arabia were more likely to occur when conditions were relatively cold and dry (4), where increasing temperature to 65°C had a strong negative effect on viral infectivity (5). Recently, the severity of COVID-19 in Europe was documented to be decreased significantly between March and May, and the seasonality of COVID-19 was suggested to explain that note (6).

Some reports suggested SARS-CoV-2 be inactivated relatively fast during summer due to the sunlight effect (7), and the overall epidemic intensity of COVID-19 was shown to be reduced slightly following days with higher temperatures (8). Short-term exposures to the ozone can influence COVID-19 transmission and initiation of the disease (9).

The COVID-19 pandemic was found to be correlated negatively with average temperature (10), wind speed 14 days ago, the temperature of the day (11), air quality (12) in terms of averaged ground levels of particulate matter concentrations (13), and relative humidity (14). Of these, temperature and humidity are essential features for predicting the COVID-19 mortality rate (15). Air pollution by an increase in PM_2.5_ accelerated transmission of SARS-CoV-2 (16) and triggered COVID-19 spread and lethality levels (17).

Nevertheless, cases in warm and humid countries have consistently increased later, opposite to the claimed effect of warm weather on the virus spread (18). On the other hand, some reports showed that there was no association between COVID-19 transmission and temperature or UV radiation in Chinese cities (19). For example, the temperature was shown to have no role in the containment of COVID-19 in Wuhan (20).

From the literature, such as dynamic multidimensional and complex weather, COVID-19 interaction cannot be explained as a general role. Still, they can suggest a regional trend that should be kept in mind when trying to understand pandemic dynamics. Based on that, we thought of exploring the correlation between weather parameters in Dubai, United Arab Emirates (UAE), and COVID-19 patients' related clinical and laboratory characteristics. To our knowledge, this paper is the first to explore this relationship in the Middle East region.

## Materials and Methods

### Patient Data Collection

A total of 434 COVID-19-positive patients admitted between January and June 2020 were recruited from Al Kuwait Hospital, Dubai, UAE. The study was approved by the Ministry of Health and Prevention (MOHAP, Research Ethics Committee number MOHAP/DXB-REC/MMM/NO. 44/2020). Adult patients (above 18 years) with COVID-19 (confirmed by nasopharyngeal polymerase chain reaction, PCR-positive sample) were enrolled. Complete current and past medical history, along with their demographic data, history of recent travel or contact with another confirmed case(s), was documented. Patients were classified according to “Clinical Management of Critically Ill COVID-19 Patients” guidelines (Version 1, April 15, 2020) issued by MOHAP (6).

### Blood and Radiological Tests

Laboratory tests were retrieved: (1) complete blood count, including neutrophil count (NR: 2–7 × 10^3^/μL), lymphocyte count (NR: 1–3 × 10^3^/μL), hemoglobin (Hb, NR: 12–15 g/dL), white cell count (WCC, NR: 4–11 × 10^3^/μL), and platelet count (NR: 150–450 × 10^3^/μL); (2) coagulation profile, including international normalized ratio (INR, NR: 0.8–1.29 s), prothrombin time (PT, NR: 9.9–12.3 s); (3) electrolytes, including sodium (Na, NR: 136–145 mmol/L) and potassium (K, NR: 3.6–5.1 mmol/L); (4) renal function tests, including urea (NR: 2.5–6.5 mmol/L), creatinine (NR: 53–88 μmol/L), and estimated glomerular filtration rate (eGFR, NR: 90–120 mL/min/1.73 m^2^); (5) liver function tests, including total serum bilirubin (NR: 3–17 μmol/L), alanine aminotransferase (ALT, NR: 16–63 IU/L), aspartate aminotransferase (AST, NR: 15–37 U/L), alkaline phosphatase (ALP, NR: 46–116 IU/L), and albumin (NR: 34–50 g/L); (6) inflammatory markers, including C-reactive protein (CRP, NR: 0–3 mg/L), D-dimers (NR: mg/dL), lactate dehydrogenase (LDH, NR: 85–227 IU/L), procalcitonin (NR: μg/L), and ferritin (8–388 μg/L). For risk of severe cases, the presence of lymphopenia, neutrophilia, high ALT and/or AST, high LDH, high CRP, high ferritin, high D-dimer, and high pro-calcitonin, above those of the age- and gender-matched references, were used as indicators of risk. Admission chest X-ray (presence of bilateral air consolidation) and computerized tomography (CT) scan (presence of bilateral peripheral ground-glass opacities) were documented.

### Climate Data

We downloaded the temperature, wind speed, cloud cover, precipitation rate, and other weather parameters of Dubai City for the duration of patient recruitment using history+ (https://www.meteoblue.com/en/historyplus), which offers immediate access to the meteoblue global weather simulation archive as shown in Table 1. We matched the date of admission for each patient with the corresponding day weather details, as shown in Table 1.

**Table 1:** The temperature, wind speed, cloud cover, precipitation rate, and other weather parameters of Dubai City for the duration of patient recruitment using history+ (https://www.meteoblue.com/en/historyplus).

**Timestamp**	**Variable**	**Unit**	**Level**	**Resolution**	**Aggregation**
Temperature [2 m elevation corrected]	Temperature	°C	2 m elevation corrected	Daily	Minimum
Temperature [2 m elevation corrected]	Temperature	°C	2 m elevation corrected	Daily	Maximum
Temperature [2 m elevation corrected]	Temperature	°C	2 m elevation corrected	Daily	Mean
Relative humidity [2 m]	Relative humidity	%	2 m	Daily	Minimum
Relative humidity [2 m]	Relative humidity	%	2 m	Daily	Maximum
Relative humidity [2 m]	Relative humidity	%	2 m	Daily	Mean
Precipitation total	Precipitation total, mm	mm	sfc	Daily	Summation
Cloud Cover Total	Cloud cover total	%	sfc	Daily	Mean
Sunshine duration	Sunshine duration	min	sfc	Daily	Summation
Shortwave radiation	Shortwave radiation	W/m^2^	sfc	Daily	Summation
Direct shortwave radiation	Direct shortwave radiation	W/m^2^	sfc	Daily	Summation
Evapotranspiration	Evapotranspiration	mm	sfc	Daily	Summation
Wind speed [10 m]	Wind speed	km/h	10 m	Daily	Minimum
Wind speed [10 m]	Wind speed	km/h	10 m	Daily	Maximum
Wind speed [10 m]	Wind speed	km/h	10 m	Daily	Mean
Wind direction dominant [10 m]	Wind direction dominant	°	10 m	Daily	None
Temperature [1,000 mb]	Temperature	°C	1,000 mb	Daily	Minimum
Temperature [1,000 mb]	Temperature	°C	1,000 mb	Daily	Maximum
Temperature [1,000 mb]	Temperature	°C	1,000 mb	Daily	Mean
Temperature	Temperature	°C	sfc	Daily	Minimum
Temperature	Temperature	°C	sfc	Daily	Maximum
Temperature	Temperature	°C	sfc	Daily	Mean

*°C, degrees Celsius; W/m^2^, watt per square meter; km/h, kilometer per hour; mm, millimeters; sfc, surface temperature*.

### Statistical Analysis

For all statistical analyses and tests, SPSS was used (IBM SPSS Statistics for Windows, Version 26.0, released 2019, IBM Corp., Armonk, NY). The chi-square test of independence was used to examine the association between categorical variables. Pearson's correlation coefficient was used to measure the correlation between different variables where correlation is significant at the 0.01 and 0.05 levels (two-tailed).

## Results

### The Clinical Severity of COVID-19 Was Significantly Dependent on the Temperature on the Day of Admission

Comparing the weather parameters on the day of admission between patients with different COVID-19 severity levels or those who needed ICU admission showed that daily temperature (°C) at 2 m elevation was the most profound factor that is statically different between patients with different COVID-19 severity levels as shown in Table 2. Patients who had severe and critical case of the disease and those who needed ICU were admitted on days with higher temperatures (23.4 ± 3.77, 23.57 ± 3.59, and 23.14 ± 3.74, *p* = 0.02). Those who showed a mild to moderate course were admitted in days with lower temperatures (20.28 ± 4.07) compared to the rest (23.51 ± 3.69).

**Table 2:** Difference in day-of-admission weather variables in Dubai City between COVID-19 patients' clinical outcomes divided into those with risk factors to develop severe COVID-19 and those without (A: old age, B: DM, C: HTN, D: CVD, or E: chronic lung disease).

**Weather variable**	**No**				**Yes**			
	**Mild to moderate (Yes/No)**	**Severe (Yes/No)**	**Critical (Yes/No)**	**ICU** **(Yes/No)**	**Mild to moderate (Yes/No)**	**Severe (Yes/No)**	**Critical (Yes/No)**	**ICU** **(Yes/No)**
**(A) Risk factors for severe illness (yes/no): Old age**								
Temperature °C 2 m elevation corrected daily minimum	0.000	0.000	0.001	0.017	0.002	0.041	ns	ns
Temperature °C 2 m elevation corrected daily maximum	0.000	0.000	0.000	0.016	0.024	ns	ns	ns
Temperature °C 2 m elevation corrected daily mean	0.000	0.000	0.000	0.008	0.006	ns	ns	ns
Relative humidity % 2 m daily minimum	0.000	0.000	0.019		0.046	ns	ns	ns
Relative humidity % 2 m daily maximum	0.000	0.037	0.000	0.002	0.004	0.004	ns	ns
Relative humidity % 2 m daily mean	0.000	0.002	0.000	0.018	0.003	0.009	ns	ns
Precipitation total mm sfc daily summation	0.012	ns	ns	ns	ns	ns	ns	ns
Cloud cover total % sfc daily mean	0.001	0.017	ns	ns	ns	ns	ns	ns
Sunshine duration min sfc daily summation	0.007	0.025	ns	ns	ns	ns	ns	ns
Shortwave radiation W/m^2^ sfc daily summation	0.000	0.000	ns	ns	ns	ns	ns	ns
Direct shortwave radiation W/m^2^ sfc daily summation	0.000	0.002	ns	ns	ns	ns	ns	ns
Evapotranspiration mm sfc daily summation	0.048	ns	ns	ns	ns	ns	ns	ns
Wind speed km/h 10 m daily minimum	ns	ns	ns	ns	ns	ns	ns	ns
Wind speed km/h 10 m daily maximum	ns	ns	ns	ns	ns	ns	ns	ns
Wind speed km/h 10 m daily mean	ns	ns	ns	ns	ns	ns	ns	ns
Wind direction dominant ° 10 m daily none	0.002	0.012	ns	ns	0.003	0.043	ns	ns
Temperature °C 1,000 mb daily minimum	0.000	0.000	0.000	0.017	0.002	ns	ns	ns
Temperature °C 1,000 mb daily maximum	0.000	0.000	0.000	0.016	0.025	ns	ns	ns
Temperature °C 1,000 mb daily mean	0.000	0.000	0.000	0.006	0.005	ns	ns	ns
Temperature °C sfc daily minimum	0.000	0.003	0.001	0.008	0.026	0.038	ns	ns
Temperature °C sfc daily maximum	0.000	0.000	0.000	0.041	ns	ns	ns	ns
Temperature °C sfc daily mean	0.000	0.000	0.000	0.007	0.017	ns	ns	ns
**(B) Risk factors for severe illness (yes/no): DM**								
Temperature °C 2 m elevation corrected daily minimum	0.000	0.000	0.003	0.024	0.000	ns	ns	ns
Temperature °C 2 m elevation corrected daily maximum	0.000	0.000	0.003	ns	0.000	0.008	ns	ns
Temperature °C 2 m elevation corrected daily mean	0.000	0.000	0.001	ns	0.000	0.020	ns	ns
Relative humidity % 2 m daily minimum	0.000	0.000	ns	ns	0.002	0.011	0.012	ns
Relative humidity % 2 m daily maximum	0.000	0.003	0.003	ns	0.000	ns	ns	ns
Relative humidity % 2 m daily mean	0.000	0.000	0.006	ns	0.000	ns	ns	ns
Precipitation total mm sfc daily summation	0.021	ns	ns	ns	ns	ns	ns	ns
Cloud cover total % sfc daily mean	0.015	ns	ns	ns	ns	ns	ns	ns
Sunshine duration min sfc daily summation	0.048	ns	ns	ns	ns	ns	ns	ns
Shortwave radiation W/m^2^ sfc daily summation	0.000	0.001	ns	ns	ns	ns	ns	ns
Direct shortwave radiation W/m^2^ sfc daily summation	0.000	0.008	ns	ns	ns	ns	ns	ns
Evapotranspiration mm sfc daily summation	ns	ns	ns	ns	ns	ns	ns	ns
Wind speed km/h 10 m daily minimum	ns	ns	ns	ns	0.036	ns	ns	ns
Wind speed km/h 10 m daily maximum	ns	ns	ns	ns	ns	ns	ns	ns
Wind speed km/h 10 m daily mean	ns	ns	ns	ns	ns	ns	ns	ns
Wind direction dominant ° 10 m daily none	0.000	0.007	ns	0.028	ns	ns	ns	ns
Temperature °C 1,000 mb daily minimum	0.000	0.000	0.000	0.032	0.000	ns	ns	ns
Temperature °C 1,000 mb daily maximum	0.000	0.000	0.005	ns	0.000	0.006	ns	ns
Temperature °C 1,000 mb daily mean	0.000	0.000	0.001	ns	0.000	0.015	ns	ns
Temperature °C sfc daily minimum	0.000	0.001	0.007	0.014	0.001		ns	ns
Temperature °C sfc daily maximum	0.000	0.000	0.009	ns	0.000	0.013	ns	ns
Temperature °C sfc daily mean	0.000	0.000	0.001	0.048	0.000	0.023	ns	ns
**(C) Risk factors for severe illness (yes/no): HTN**								
Temperature °C 2 m elevation corrected daily minimum	0.000	0.000	0.001	0.050	0.000	ns	ns	ns
Temperature °C 2 m elevation corrected daily maximum	0.000	0.000	0.000	0.024	0.001	ns	ns	ns
Temperature °C 2 m elevation corrected daily mean	0.000	0.000	0.000	0.016	0.000	ns	ns	ns
Relative humidity % 2 m daily minimum	0.000	0.000	0.012			ns	ns	ns
Relative humidity % 2 m daily maximum	0.000	0.016	0.000	0.001	0.003	ns	ns	ns
Relative humidity % 2 m daily mean	0.000	0.000	0.000	0.003	0.013	ns	ns	ns
Precipitation total mm sfc daily summation	0.012	ns	ns	ns	ns	ns	ns	ns
Cloud cover total % sfc daily mean	0.007	0.036	ns	ns	ns	ns	ns	ns
Sunshine duration min sfc daily summation	0.028	0.043	ns	ns	ns	ns	0.045	ns
Shortwave radiation W/m^2^ sfc daily summation	0.000	0.000	ns	ns	ns	ns	ns	ns
Direct shortwave radiation W/m^2^ sfc daily summation	0.000	0.002	ns	ns	ns	ns	ns	ns
Evapotranspiration mm sfc daily summation	ns	ns	ns	ns	ns	ns	ns	ns
Wind speed km/h 10 m daily minimum	ns	ns	ns	ns	ns	ns	ns	ns
Wind speed km/h 10 m daily maximum	ns	ns	ns	ns	ns	ns	ns	ns
Wind speed km/h 10 m daily mean	ns	ns	ns	ns	ns	ns	ns	ns
Wind direction dominant ° 10 m daily none	0.000	0.000	0.000	ns	ns	ns	ns	ns
Temperature °C 1,000 mb daily minimum	0.000	0.000	0.000	0.044	0.000	ns	0.035	ns
Temperature °C 1,000 mb daily maximum	0.000	0.000	0.000	0.022	0.002	ns	ns	ns
Temperature °C 1,000 mb daily mean	0.000	0.000	0.003	0.012	0.000	ns	ns	ns
Temperature °C sfc daily minimum	0.000	0.001	0.001	ns	0.004	ns	ns	ns
Temperature °C sfc daily maximum	0.000	0.000	0.000	ns	0.001	ns	0.045	ns
Temperature °C sfc daily mean	0.000	0.000	ns	0.022	0.000	ns	ns	ns
**(D) Risk factors for severe illness (yes/no): CVD**								
Temperature °C 2 m elevation corrected daily minimum	0.000	0.000	0.001	ns	ns	ns	ns	ns
Temperature °C 2 m elevation corrected daily maximum	0.000	0.000	0.000	0.036	ns	ns	ns	ns
Temperature °C 2 m elevation corrected daily mean	0.000	0.000	0.000	0.024	ns	ns	ns	ns
Relative humidity % 2 m daily minimum	0.000	0.000	0.008	ns	ns	ns	ns	ns
Relative humidity % 2 m daily maximum	0.000	0.001	0.000	0.020	ns	ns	ns	ns
Relative humidity % 2 m daily mean	0.000	0.000	0.000	ns	ns	ns	ns	ns
Precipitation total mm sfc daily summation	0.015	ns	ns	ns	ns	ns	ns	ns
Cloud cover total % sfc daily mean	0.001	ns	ns	ns	ns	ns	ns	ns
Sunshine duration min sfc daily summation	0.004	ns	ns	ns	ns	ns	ns	ns
Shortwave radiation W/m^2^ sfc daily summation	0.000	0.001	0.027	ns	ns	ns	ns	ns
Direct shortwave radiation W/m^2^ sfc daily summation	0.000	0.009	ns	ns	ns	ns	ns	ns
Evapotranspiration mm sfc daily summation	ns	ns	ns	ns	ns	ns	ns	ns
Wind speed km/h 10 m daily minimum	ns	ns	ns	ns	ns	ns	ns	ns
Wind speed km/h 10 m daily maximum	ns	ns	ns	ns	ns	ns	ns	ns
Wind speed km/h 10 m daily mean	ns	ns	ns	ns	ns	ns	ns	ns
Wind direction dominant ° 10 m daily none	0.000	0.001	ns	ns	ns	ns	ns	ns
Temperature °C 1,000 mb daily minimum	0.000	0.000	0.000	0.049	ns	ns	ns	ns
Temperature °C 1,000 mb daily maximum	0.000	0.000	0.000	0.040	ns	ns	ns	ns
Temperature °C 1,000 mb daily mean	0.000	0.000	0.000	0.023	ns	ns	ns	ns
Temperature °C sfc daily minimum	0.000	0.000	0.005	ns	ns	ns	ns	ns
Temperature °C sfc daily maximum	0.000	0.000	0.000	ns	ns	ns	ns	ns
Temperature °C sfc daily mean	0.000	0.000	0.000	0.022	ns	ns	ns	ns
**(E) Risk factors for severe illness (yes/no): Chronic lung disease**								
Temperature °C 2 m elevation corrected daily minimum	0.000	0.000	0.000	0.022	0.021	ns	ns	ns
Temperature °C 2 m elevation corrected daily maximum	0.000	0.000	0.000	0.025	0.027	ns	ns	ns
Temperature °C 2 m elevation corrected daily mean	0.000	0.000	0.000	0.012	0.014	ns	ns	ns
Relative humidity % 2 m daily minimum	0.000	0.000	0.006		ns	ns	ns	ns
Relative humidity % 2 m daily maximum	0.000	0.003	0.000	0.015	ns	ns	ns	ns
Relative humidity % 2 m daily mean	0.000	0.000	0.000	0.036	ns	ns	ns	ns
Precipitation total mm sfc daily summation	0.012	ns	ns	ns	ns	ns	ns	ns
Cloud cover total % sfc daily mean	0.001	ns	ns	ns	ns	ns	ns	ns
Sunshine duration min sfc daily summation	0.006	ns	ns	ns	ns	ns	ns	ns
Shortwave radiation W/m^2^ sfc daily summation	0.000	0.001	0.031	ns	ns	ns	ns	ns
Direct shortwave radiation W/m^2^ sfc daily summation	0.000	0.006	ns	ns	ns	ns	ns	ns
Evapotranspiration mm sfc daily summation	ns	ns	ns	ns	ns	ns	0.038	0.038
Wind speed km/h 10 m daily minimum	ns	ns	ns	ns	ns	ns	ns	ns
Wind speed km/h 10 m daily maximum	ns	ns	ns	ns	ns	ns	ns	ns
Wind speed km/h 10 m daily mean	ns	ns	ns	ns	ns	ns	ns	ns
Wind direction dominant ° 10 m daily none	0.000	0.001			ns	ns	ns	ns
Temperature °C 1,000 mb daily minimum	0.000	0.000	0.000	0.026	0.029	ns	ns	ns
Temperature °C 1,000 mb daily maximum	0.000	0.000	0.000	0.027	0.021	ns	ns	ns
Temperature °C 1,000 mb daily mean	0.000	0.000	0.000	0.012	0.018	ns	ns	ns
Temperature °C sfc daily minimum	0.000	0.001	0.003	0.030	ns	ns	ns	ns
Temperature °C sfc daily maximum	0.000	0.000	0.000	0.049	ns	ns	ns	ns
Temperature °C sfc daily mean	0.000	0.000	0.000	0.012	0.016	ns	ns	ns

### Differences in Daily Admission Temperature Affect the Clinical Outcomes in Patients Who Have No Risk Factors to Develop Severe COVID-19

To delineate whether the difference in the clinical outcomes based on daily temperature was different among patients with risk factors to develop severe COVID-19 or not, we divided the patients into those with such risk factors [old age, diabetes mellitus (DM), hypertension (HTN), cardiovascular disease (CVD), or chronic lung disease] and those without. Differences in daily admission temperature affect the clinical outcomes in patients who have no risk factors to develop severe COVID-19, while patients with such risk factors showed no significant difference in the clinical outcomes as shown in Tables 3A–**E**. This showed that the effect of weather on the clinical outcomes is important in those who have no risk as such risks can affect the outcome regardless of the weather parameters.

**Table 3A:** Difference in day-of-admission weather variables in Dubai City between COVID-19 patients' clinical outcomes: Death.

**Weather variable**	**Death**				***p*-value**
	**No**		**Yes**		
	**Mean**	**SD**	**Mean**	**SD**	
Temperature °C 2 m elevation corrected daily minimum	22.24	4.22	23.09	3.50	ns
Temperature °C 2 m elevation corrected daily maximum	37	5.92	38.8	4.79	0.016
Temperature °C 2 m elevation corrected daily mean	29.01	4.89	30.46	3.98	0.02
Relative humidity % 2 m daily minimum	19	9	16	7	0.008
Relative humidity % 2 m daily maximum	72	18	66	18	0.012
Relative humidity % 2 m daily mean	45.31	14.17	40.15	13.93	0.005
Precipitation total mm sfc daily summation	0.305	1.60	0.11	0.35	ns
Cloud cover total % sfc daily mean	22.29	25.77	16.12	22.08	ns
Sunshine duration min sfc daily summation	615.2	199.96	657.4	165.95	ns
Shortwave radiation W/m^2^ sfc daily summation	6,933	1266.91	7296	954.79	0.023
Direct shortwave radiation W/m^2^ sfc daily summation	4433	1077.42	4703	859.03	0.047
Evapotranspiration mm sfc daily summation	0.348	0.24	0.369	0.19	ns
Wind speed km/h 10 m daily minimum	2.788	2.62	3.306	3.04	ns
Wind speed km/h 10 m daily maximum	15.44	4.66	15.28	5.39	ns
Wind speed km/h 10 m daily mean	8.312	3.30	8.471	3.78	ns
Wind direction dominant ° 10 m daily none	196.1	114.62	189.5	106.88	ns
Temperature °C 1,000 mb daily minimum	24.17	5.73	25.65	5.02	0.043
Temperature °C 1,000 mb daily maximum	34.72	5.77	36.45	4.77	0.018
Temperature °C 1,000 mb daily mean	29.08	5.68	30.85	4.80	0.014
Temperature °C sfc daily minimum	19.9	4.01	20.54	3.65	ns
Temperature °C sfc daily maximum	44.81	6.76	46.71	5.34	0.026
Temperature °C sfc daily mean	30.71	4.91	32.12	3.97	0.024

### The Clinical Outcomes (Death and Organ Failure) of COVID-19 Were Significantly Dependent on the Day-of-Admission Temperature and Relative Humidity

The next step was to compare the weather parameters of the day of admission between different COVID-19 patients who developed outcomes and complications (like death, acute cardiac injury, acute kidney injury, acute liver injury, acidosis, and septic shock) and those who did not develop such complications.

#### Death

**Figure 1:**
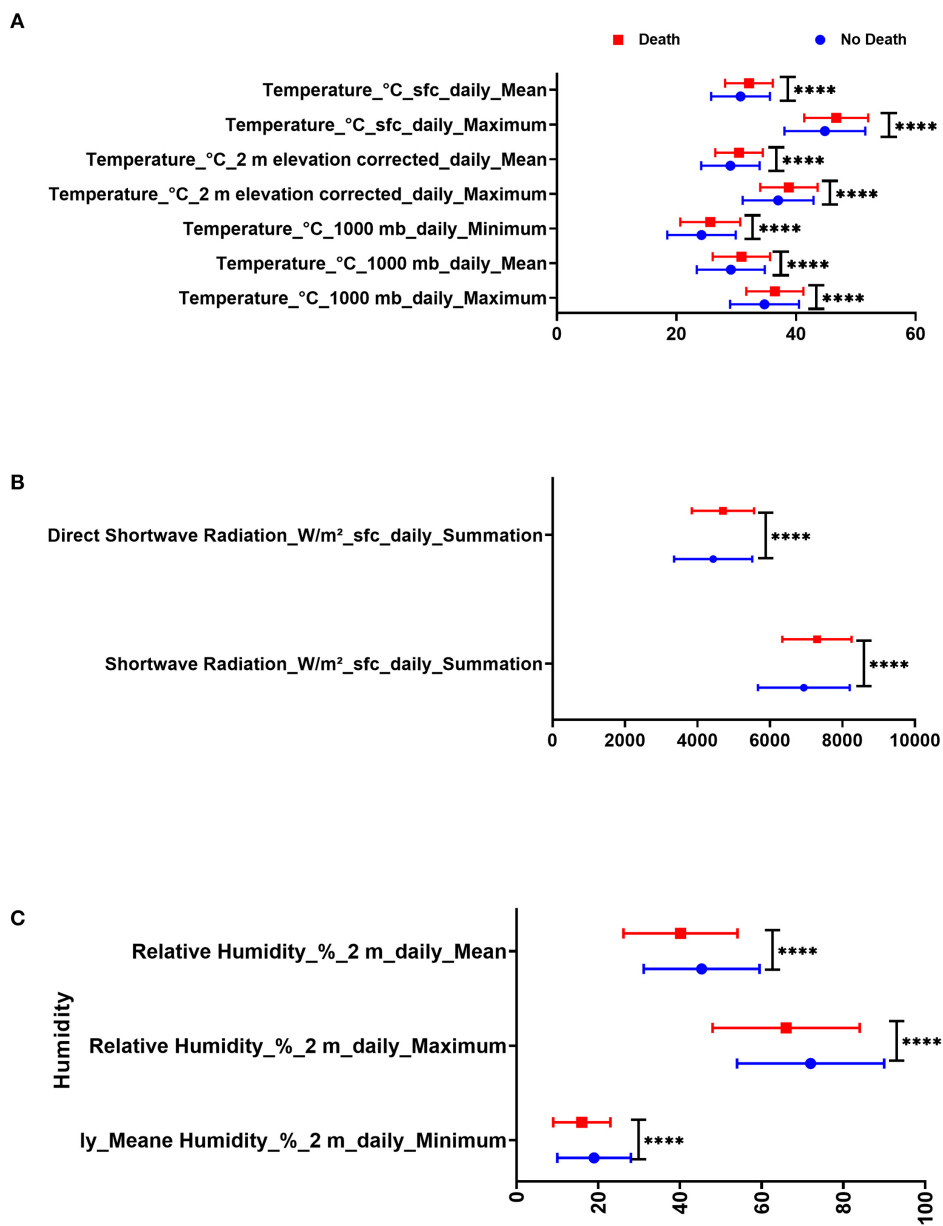
Difference in day admission weather variables in Dubai City between COVID-19 patients with death and those who were discharged in terms of **(A)** temperature, **(B)** humidity, and **(C)** radiation.

There were significant statistical differences in the mean of temperature, relative humidity, shortwave radiation, and direct shortwave radiation between the group of COVID-19 patients who died and the group of COVID-19 patients who survived. COVID-19 patients admitted in days with higher temperatures, higher solar radiation, and less humidity were at higher risk of death, as shown in Figure 1.

COVID-19 patients who died due to the disease were admitted on days with less relative humidity (40.15 ± 13.93% 2 m daily mean) compared to those who survived (45.31 ± 14.17% 2 m daily mean, *p* = 0.005). Also, COVID-19 patients who died due to the disease were admitted on days with higher temperature (32.12 ± 3.97°C sfc daily mean) compared to those who survived (30.71 ± 4.91°C sfc daily mean, *p* =0.024) as shown in Table 3A. COVID-19 patients were divided according to mortality and were compared in terms of day-of-admission weather parameters.

#### Acute Cardiac Injury

COVID-19 patients who developed acute cardiac injury were admitted on days with higher temperature (30.77 ± 3.89°C 2 m elevation corrected daily mean) compared to patients without cardiac injury (28.81 ± 4.93°C 2 m elevation corrected daily mean, *p* ≤ 0.005) as shown in Table 3B. On the other hand, COVID-19 patients who developed acute cardiac injury were admitted on days with less relative humidity (41.94 ± 2.99% 2 m daily mean) compared to the rest of the patients (45.19 ± 14.53% 2 m daily mean, *p* = 0.048).

**Table 3B:** Difference in day-of-admission weather variables in Dubai City between COVID-19 patients' clinical outcomes: Acute cardiac injury.

**Weather variable**	**Acute cardiac injury**				***p*-value**
	**No**		**Yes**		
	**Mean**	**SD**	**Mean**	**SD**	
Temperature °C 2 m elevation corrected daily minimum	22.08	4.24	23.43	3.49	0.004
Temperature °C 2 m elevation corrected daily maximum	36.75	5.96	39.17	4.73	0
Temperature °C 2 m elevation corrected daily mean	28.81	4.93	30.77	3.89	0
Relative humidity % 2 m daily minimum	19.00	9.00	17.00	7.00	0.014
Relative humidity % 2 m daily maximum	72.00	18.00	68.00	18.00	ns
Relative humidity % 2 m daily mean	45.19	14.53	41.94	12.99	0.048
Precipitation total mm sfc daily summation	0.32	1.66	0.10	0.36	ns
Cloud cover total % sfc daily mean	22.39	25.78	17.45	23.22	ns
Sunshine duration min sfc daily summation	614.48	197.32	648.59	186.44	ns
Shortwave radiation W/m^2^ sfc daily summation	6903.76	1268.17	7298.58	1023.49	0.005
Direct shortwave radiation W/m^2^ sfc daily summation	4409.61	1074.61	4711.49	920.49	0.012
Evapotranspiration mm sfc daily summation	0.36	0.24	0.33	0.20	ns
Wind speed km/h 10 m daily minimum	2.79	2.63	3.17	2.92	ns
Wind speed km/h 10 m daily maximum	15.41	4.66	15.45	5.22	ns
Wind speed km/h 10 m daily mean	8.31	3.34	8.45	3.56	ns
Wind direction dominant ° 10 m daily none	194.25	115.45	197.88	106.02	ns
Temperature °C 1,000 mb daily minimum	23.98	5.77	25.90	4.90	0.003
Temperature °C 1,000 mb daily maximum	34.49	5.80	36.79	4.70	0
Temperature °C 1,000 mb daily mean	28.87	5.74	31.09	4.64	0.001
Temperature °C sfc daily minimum	19.74	4.05	20.93	3.46	0.008
Temperature °C sfc daily maximum	44.50	6.74	47.26	5.53	0
Temperature °C sfc daily mean	30.48	4.93	32.55	3.94	0

#### Acute Kidney Injury

COVID-19 patients who showed acute kidney injury were admitted on days with higher temperature (30.42 ± 3.97°C 2 m elevation corrected daily mean) compared to patients with intact kidney (28.95 ± 4.92°C 2 m elevation corrected daily mean, *p* = 0.01) as shown in Table 3C.

**Table 3C:** Difference in day-of-admission weather variables in Dubai City between COVID-19 patients' clinical outcomes: Acute kidney injury.

**Weather variable**	**Acute kidney injury**				***p*-value**
	**No**		**Yes**		
	**Mean**	**SD**	**Mean**	**SD**	
Temperature °C 2 m elevation corrected daily minimum	22.18	4.23	23.20	3.57	0.038
Temperature °C 2 m elevation corrected daily maximum	36.95	5.97	38.64	4.83	0.015
Temperature °C 2 m elevation corrected daily mean	28.95	4.92	30.42	3.97	0.01
Relative humidity % 2 m daily minimum	19.00	9.00	18.00	7.00	ns
Relative humidity % 2 m daily maximum	72.00	18.00	67.00	18.00	ns
Relative humidity % 2 m daily mean	45.11	14.37	41.90	13.52	ns
Precipitation total mm sfc daily summation	0.31	1.64	0.14	0.42	ns
Cloud cover total % sfc daily mean	21.96	25.70	18.59	23.52	ns
Sunshine duration min sfc daily summation	618.12	196.51	638.05	190.41	ns
Shortwave radiation W/m^2^ sfc daily summation	6944.16	1250.10	7183.31	1120.31	ns
Direct shortwave radiation W/m^2^ sfc daily summation	4443.32	1058.58	4612.11	1002.74	ns
Evapotranspiration mm sfc daily summation	0.35	0.24	0.36	0.21	ns
Wind speed km/h 10 m daily minimum	2.84	2.66	2.99	2.88	ns
Wind speed km/h 10 m daily maximum	15.44	4.71	15.33	5.11	ns
Wind speed km/h 10 m daily mean	8.31	3.35	8.45	3.53	ns
Wind direction dominant ° 10 m daily none	193.67	114.86	200.58	107.30	ns
Temperature °C 1,000 mb daily minimum	24.15	5.78	25.46	4.95	ns
Temperature °C 1,000 mb daily maximum	34.68	5.80	36.31	4.79	0.016
Temperature °C 1,000 mb daily mean	29.03	5.72	30.71	4.77	0.012
Temperature °C sfc daily minimum	19.81	4.03	20.76	3.57	0.046
Temperature °C sfc daily maximum	44.77	6.78	46.50	5.54	0.029
Temperature °C sfc daily mean	30.64	4.95	32.13	3.96	0.01

#### Acute Liver Injury

COVID-19 patients who showed acute liver injury were admitted on days with higher temperature (30.78 ± 3.89°C 2 m elevation corrected daily mean) compared to patients with intact liver (28.88 ± 4.90°C 2 m elevation corrected daily mean, *p* = 0.001) as shown in Table 3D.

**Table 3D:** Difference in day-of-admission weather variables in Dubai City between COVID-19 patients' clinical outcomes: Acute liver injury.

**Weather variable**	**Acute liver injury**				***p*-value**
	**No**		**Yes**		
	**Mean**	**SD**	**Mean**	**SD**	
Temperature °C 2 m elevation corrected daily minimum	22.05	4.24	23.74	3.29	0.001
Temperature °C 2 m elevation corrected daily maximum	36.90	5.92	38.93	4.93	0.004
Temperature °C 2 m elevation corrected daily mean	28.88	4.90	30.78	3.89	0.001
Relative humidity % 2 m daily minimum	19.00	9.00	18.00	8.00	ns
Relative humidity % 2 m daily maximum	71.00	18.00	69.00	17.00	ns
Relative humidity % 2 m daily mean	44.85	14.42	42.86	13.47	ns
Precipitation total mm sfc daily summation	0.31	1.64	0.12	0.37	ns
Cloud cover total % sfc daily mean	21.19	24.52	21.67	28.41	ns
Sunshine duration min sfc daily summation	623.31	188.12	617.18	223.62	ns
Shortwave radiation W/m^2^ sfc daily summation	6958.65	1199.60	7131.62	1336.45	ns
Direct shortwave radiation W/m^2^ sfc daily summation	4462.15	1008.79	4539.91	1204.90	ns
Evapotranspiration mm sfc daily summation	0.36	0.24	0.30	0.19	0.021
Wind speed km/h 10 m daily minimum	2.80	2.56	3.18	3.24	ns
Wind speed km/h 10 m daily maximum	15.24	4.57	16.13	5.57	ns
Wind speed km/h 10 m daily mean	8.22	3.20	8.84	4.04	ns
Wind direction dominant ° 10 m daily none	192.74	114.09	204.72	110.08	ns
Temperature °C 1,000 mb daily minimum	23.97	5.75	26.27	4.74	0.001
Temperature °C 1,000 mb daily maximum	34.64	5.76	36.55	4.90	0.005
Temperature °C 1,000 mb daily mean	28.96	5.71	31.07	4.67	0.002
Temperature °C sfc daily minimum	19.71	4.02	21.25	3.45	0.001
Temperature °C sfc daily maximum	44.71	6.67	46.82	5.94	0.008
Temperature °C sfc daily mean	30.57	4.93	32.52	3.87	0.001

#### Acidosis

COVID-19 patients who end up with acidosis were admitted on days with higher temperature (30.62 ± 4.07°C 2 m elevation corrected daily mean) compared to patients who did not develop acidosis (28.90 ± 4.89°C 2 m elevation corrected daily mean, *p* = 0.003) as shown in Table 3E.

**Table 3E:** Difference in day-of-admission weather variables in Dubai City between COVID-19 patients' clinical outcomes: Acidosis.

**Weather variable**	**Acidosis**				***p*-value**
	**No**		**Yes**		
	**Mean**	**SD**	**Mean**	**SD**	
Temperature °C 2 m elevation corrected daily minimum	22.18	4.26	23.17	3.42	0.043
Temperature °C 2 m elevation corrected daily maximum	36.84	5.87	39.08	5.13	0.001
Temperature °C 2 m elevation corrected daily mean	28.90	4.89	30.62	4.07	0.003
Relative humidity % 2 m daily minimum	19.00	9.00	17.00	8.00	ns
Relative humidity % 2 m daily maximum	71.00	18.00	69.00	18.00	ns
Relative humidity % 2 m daily mean	44.93	14.30	42.64	13.98	ns
Precipitation total mm sfc daily summation	0.31	1.64	0.11	0.37	ns
Cloud cover total % sfc daily mean	22.31	26.05	17.26	21.73	ns
Sunshine duration min sfc daily summation	613.93	201.00	654.23	168.03	ns
Shortwave Radiation W/m^2^ sfc daily summation	6904.68	1262.54	7335.36	1015.49	0.003
Direct shortwave radiation W/m^2^ sfc daily summation	4412.80	1072.20	4729.86	913.11	0.011
Evapotranspiration mm sfc daily summation	0.36	0.24	0.33	0.21	ns
Wind speed km/h 10 m daily minimum	2.79	2.60	3.18	3.05	ns
Wind speed km/h 10 m daily maximum	15.40	4.62	15.49	5.41	ns
Wind speed km/h 10 m daily mean	8.27	3.28	8.62	3.79	ns
Wind direction dominant ° 10 m daily none	193.93	115.31	199.48	105.55	ns
Temperature °C 1,000 mb daily minimum	24.03	5.75	25.90	4.94	0.005
Temperature °C 1,000 mb daily maximum	34.57	5.70	36.70	5.12	0.002
Temperature °C 1,000 mb daily mean	28.96	5.68	30.97	4.87	0.002
Temperature °C sfc daily minimum	19.80	4.05	20.79	3.47	0.037
Temperature °C sfc daily maximum	44.60	6.66	47.17	5.85	0.001
Temperature °C sfc daily mean	30.56	4.90	32.44	4.07	0.001

#### Septic Shock

Interestingly, COVID-19 patients with septic shock were admitted on days with higher temperature (30.73 ± 3.96°C 2 m elevation corrected daily mean) compared to patients without septic shock (28.96 ± 4.88°C 2 m elevation corrected daily mean, *p* = 0.005), as shown in Table 3F. Again, COVID-19 patients who developed septic shock were admitted on days with less relative humidity (40.63 ± 13.32% 2 m daily mean) compared to the rest of the patients (45.20 ± 14.32 2 m daily mean, *p* = 0.014).

**Table 3F:** Difference in day-of-admission weather variables in Dubai City between COVID-19 patients' clinical outcomes: Septic shock.

**Weather variable**	**Septic Shock**				***p*-value**
	**No**		**Yes**		
	**Mean**	**SD**	**Mean**	**SD**	
Temperature °C 2 m elevation corrected daily minimum	22.20	4.20	23.34	3.58	0.033
Temperature °C 2 m elevation corrected daily maximum	36.95	5.91	39.09	4.80	0.004
Temperature °C 2 m elevation corrected daily mean	28.96	4.88	30.73	3.96	0.005
Relative humidity % 2 m daily minimum	19.00	9.00	16.00	7.00	0.01
Relative humidity % 2 m daily maximum	71.00	18.00	67.00	18.00	0.05
Relative humidity % 2 m daily mean	45.20	14.32	40.63	13.32	0.014
Precipitation total mm sfc daily summation	0.30	1.60	0.14	0.42	ns
Cloud cover total % sfc daily mean	22.44	25.84	15.27	21.35	0.03
Sunshine duration min sfc daily summation	614.95	200.15	659.34	163.76	ns
Shortwave radiation W/m^2^ sfc daily summation	6933.76	1262.12	7295.28	983.52	0.024
Direct shortwave radiation W/m^2^ sfc daily summation	4434.43	1072.42	4699.21	889.63	ns
Evapotranspiration mm sfc daily summation	0.35	0.24	0.34	0.20	ns
Wind speed km/h 10 m daily minimum	2.79	2.60	3.31	3.16	ns
Wind speed km/h 10 m daily maximum	15.41	4.62	15.45	5.62	ns
Wind speed km/h 10 m daily mean	8.28	3.28	8.65	3.92	ns
Wind direction dominant ° 10 m daily none	195.87	114.88	190.85	105.41	ns
Temperature °C 1,000 mb daily minimum	24.09	5.71	26.10	4.98	0.006
Temperature °C 1,000 mb daily maximum	34.68	5.75	36.72	4.80	0.005
Temperature °C 1,000 mb daily mean	29.02	5.66	31.16	4.77	0.003
Temperature °C sfc daily minimum	19.86	3.99	20.76	3.69	ns
Temperature °C sfc daily maximum	44.75	6.72	47.05	5.43	0.007
Temperature °C sfc daily mean	30.67	4.90	32.38	3.97	0.006

### Higher Temperature, Less Humidity, and More Radiation on Admission Dates Were Associated With Specific Laboratory Markers

The next step was to find the correlation between the three weather measurements that were found to be significantly different between the group of COVID-19 that had death as an outcome and the group that survived COVID-19; namely, the weather parameters were high temperature, less humidity, and more radiation. The weather parameters were correlated with patients' clinical and laboratory parameters. The results showed that higher temperature, less humidity, and more radiation on admissions dates were associated with higher CRP, neutrophil count, age at diagnosis, WCC, AST, and ALP but lower lymphocyte count, eGFR, Hb, Na, and albumin, as shown in Table 4.

**Table 4:** Correlation between the significant weather variables (temperature, humidity, and radiation) on the day of admission with patients' clinical and laboratory parameters.

**Variables**		**Temperature °C 2 m elevation corrected daily mean**			**Relative humidity % 2 m daily mean**			**Direct shortwave radiation W/m^**2**^ sfc daily summation**			**Temperature °C sfc daily mean**	
	**Pearson correlation**	**Sig. (2-tailed)**	**N**	**Pearson correlation**	**Sig. (2-tailed)**	**N**	**Pearson correlation**	**Sig. (2-tailed)**	**N**	**Pearson correlation**	**Sig. (2-tailed)**	**N**
CRP	0.258^**^	0	429	−0.203^**^	0	429	0.125^**^	0.01	429	0.260^**^	0	429
Neutrophil count	0.205^**^	0	433	−0.175^**^	0	433	0.044	0.356	433	0.194^**^	0	433
Age at diagnosis	0.134^**^	0.005	433	−0.066	0.168	433	0.146^**^	0.002	433	0.148^**^	0.002	433
WCC	0.131^**^	0.006	433	−0.130^**^	0.007	433	0.035	0.464	433	0.126^**^	0.009	433
AST	0.128^**^	0.008	433	−0.038	0.43	433	0.052	0.281	433	0.137^**^	0.004	433
ALP	0.111^*^	0.021	433	−0.09	0.06	433	0.064	0.186	433	0.112^*^	0.019	433
Duration of illness, days (symptom onset to admission)	0.058	0.225	433	−0.099^*^	0.04	433	0.084	0.082	433	0.054	0.259	433
Lymphocyte count	−0.104^*^	0.031	433	0.081	0.093	433	−0.061	0.203	433	−0.118^*^	0.014	433
eGFR	−0.144^**^	0.003	432	0.066	0.173	432	−0.128^**^	0.008	432	−0.152^**^	0.002	432
Hb	−0.162^**^	0.001	433	0.120^*^	0.012	433	−0.107^*^	0.026	433	−0.161^**^	0.001	433
Na	−0.170^**^	0	433	0.07	0.143	433	−0.119^*^	0.013	433	−0.177^**^	0	433
Albumin	−0.267^**^	0	432	0.258^**^	0	432	−0.166^**^	0.001	432	−0.263^**^	0	432

** *Correlation is significant at the 0.01 level (two-tailed)*.

* *Correlation is significant at the 0.05 level (two-tailed)*.

## Discussion

High temperatures create a substantial health burden (21) and pose significant public health concerns worldwide, like increased premature deaths attributable to either heat or cold in selected countries (22), but such a burden is not always associated with extreme (high or low) temperatures due to the complexity of weather-related health effects (23). Using the spatial synoptic classification, which uses the combined effect of meteorological factors rather than temperature only for assessing the weather effects on health, is more appropriate to delineate the link between weather and health-related issues (24). For that reason, we explored the different meteorological factors on the outcome of COVID-19 patients in our locally recruited cohort.

Our results showed that in our cohort, COVID-19 patients who were admitted in days with higher temperature, higher solar radiation, and less humidity had a higher chance to develop severe and critical COVID-19, to need ICU admission, and to die than those who were admitted in days with lower temperature and higher relative humidity.

Our results showed also that higher temperature and less humidity were associated with devastating consequences of COVID-19 like acute cardiac, liver and kidney injuries, increased acidosis, and septic shock. All these can explain the positive correlation between higher temperature and less humidity with increased mortality on our cohort. In confirmation to such association, our finding of higher temperature, higher solar radiation, and less humidity association with higher deaths can be linked to the association with markers known to be associated with poor prognosis in COVID-19 patients like higher CRP, neutrophil count, WCC, AST, and ALP and lower lymphocyte count, eGFR, Hb, Na, and albumin.

But it is not easy to prove causality in such an observational study where many confounding factors might play different roles in the clinical outcomes (6), and that is why we looked for the effect of known risk factors among our cohort to measure the real effect of weather on the outcomes. Interestingly, we found that the effect of weather was more obvious in patients who have no history of risk factors to develop severe COVID-19 as such patients with risk factors showed no difference in their outcomes in different weather conditions. This might indicate the possible direct effect of weather on the consequences of the COVID-19 course. This finding goes with the earlier findings where temperature variation and humidity were found to be important factors affecting COVID-19 mortality (25). Recently, researchers found that there is an observed decrease in COVID-19 severity with higher outside temperature, which was explained by restoration of impaired mucosal barrier function due to dry air (6). Substantial community outbreaks of COVID-19 were found to show some preferred latitude, temperature, and humidity measurements, same as other seasonal respiratory virus (26). Climatic factors were thought to affect COVID-19 incidence and severity and can be used in preventive and public health actions against upcoming outbreaks of the disease (27).

So, one can postulate that our finding of more severe COVID-19 with lower relative humidity and higher temperature is due to impaired mucosal defense that aids the virus in its infection and propagation. Effective humidity of inhaled air can modulate hydration of the respiratory epithelium that boosts mucosal immunity, so lower relative humidity and higher temperature can actually decrease mucosal hydration and impair it in return (28).

It was shown earlier that the rate of cases presenting daily was inversely associated with daily temperature; such a rate was decreased on days when the temperature was above 52°F, 5 days earlier (29), as for every 1°C increase in temperature, daily new cases of COVID-19 were reduced by 3.08% (30). So, in our case, the higher temperature might decrease the daily reported cases, but the cases that were admitted despite unfavorable weather might be exposed to a higher dose of the virus with closer contact, which can explain the worse course outcome.

The relation between high temperature and mortality in the general population was documented in some reports as it was associated with increased mortality risk, particularly in females and adults aged 20–59 years (31). High air temperature in early summer was associated with increased mortality compared with that in late summer (32), where apparent temperature appeared to be the most critical predictor of heat-related mortality for all-cause mortality (33). On the other hand, the effects of cold on all-cause mortality were highest among people over 75 years old, mainly due to myocardial infarction, ischemic heart diseases, and respiratory diseases (34). Older adults with medical and psychiatric conditions without home heat are most at risk of death due to hypothermia (35).

Patients with chronic diseases may have impaired thermoregulatory ability (36). SARS-CoV-2 is known to increase the hypothalamic–thermoregulatory set, which adversely impacts the outcomes and mortality in patients with COVID-19 (37). Short-term exposure to weather-related stimuli like heat is associated with increased glucocorticoid level that serves as a physiological mechanism promoting fitness during inclement weather, but this, if extended, might have an adverse effect (38). Hot weather in disturbed thermoregulatory conditions induced by SARS-CoV-2 might aggravate mortality.

Heat stress was shown to increase a more significant percentage of neutrophils and a lesser percentage of lymphocytes in animals (39). In humans, heat stress conditions were shown to reduce leukocyte levels, and immunoglobulin concentration can weaken the immune system (40). Elevated ambient temperatures can affect the cardiocirculatory and hormonal systems, resulting in changes in neutrophil and monocyte cell trafficking (41). The neutrophil-to-lymphocyte ratio, interleukin (IL)-6, IL-1β, and CRP were higher in persons exposed more frequently to heat per month, which might predispose to systemic inflammation (42). Long-term heat exposure was found to enhance chemokines to recruitment neutrophils to the lungs, leading to an increased risk of respiratory illnesses (43). However, those recruited neutrophils might have impaired phagocytosis and reactive oxygen species (ROS) production under severely high temperatures, leading to a higher occurrence of infections during hot weather (44). Our finding of a correlation between higher temperature and lower eGFR and old age during a presentation can be explained by the fact that older age in men and women exposed to short-term ambient temperature was significantly associated with kidney injury biomarkers (45).

In conclusion, our study highlighted the importance of taking weather-related variables into account to understand the dynamics of mortality or clinical outcomes in COVID-19 patients in countries with hot climates like the UAE. The effect of hot stress might weaken the immune system and unleash an inflammatory response that makes some people, especially those with comorbidities or those who are older, more susceptible to infections or to develop aggressive inflammation that ends up with complications and mortality.

## Data Availability Statement

The original contributions generated for this study are included in the article/supplementary material, further inquiries can be directed to the corresponding author/s.

## Ethics Statement

The studies involving human participants were reviewed and approved by Ministry of Health and Prevention (MOHAP) Research Ethics Committee number (MOHAP/DXB-REC/MMM/NO.44/2020).

## Consent for Publication

All authors have agreed to the publication and to be accountable for all aspects of the work in ensuring that questions related to the accuracy or integrity of any part of the work are appropriately investigated and resolved.

## Author Contributions

All authors listed have made a substantial, direct and intellectual contribution to the work, and approved it for publication.

## Conflict of Interest

The authors declare that the research was conducted in the absence of any commercial or financial relationships that could be construed as a potential conflict of interest.

## References

[1] Gupta S Raghuwanshi GS Chanda A. Effect of weather on COVID-19 spread in the US: a prediction model for India in 2020. Sci Total Environ. (2020) 728:138860. 10.1016/j.scitotenv.2020.138860 32334160 PMC7194548

[2] Adedokun KA Olarinmoye AO Mustapha JO Kamorudeen RT. A close look at the biology of SARS-CoV-2, and the potential influence of weather conditions and seasons on COVID-19 case spread. Infect Dis Poverty. (2020) 9:77. 10.1186/s40249-020-00688-1 32586369 PMC7316581

[3] Raina SK Kumar R Bhota S Gupta G Kumar D Chauhan R. Does temperature and humidity influence the spread of Covid-19?: a preliminary report. J Family Med Prim Care. (2020) 9:1811–4. 10.4103/jfmpc.jfmpc_494_20 32670923 PMC7346968

[4] Gardner EG Kelton D Poljak Z Van Kerkhove M von Dobschuetz S Greer AL. A case-crossover analysis of the impact of weather on primary cases of Middle East respiratory syndrome. BMC Infect Dis. (2019) 19:113. 10.1186/s12879-019-3729-5 30717685 PMC6362578

[5] Leclercq I Batéjat C Burguière AM Manuguerra JC. Heat inactivation of the Middle East respiratory syndrome coronavirus. Influenza Other Respir Viruses. (2014) 8:585–6. 10.1111/irv.12261 25074677 PMC4181824

[6] Kifer D Bugada D Villar-Garcia J Gudelj I Menni C Sudre C. Effects of environmental factors on severity and mortality of COVID-19. medRxiv [Preprint]. (2020). 10.1101/2020.07.11.20147157 33553204 PMC7855590

[7] Sagripanti JL Lytle CD. Estimated Inactivation of Coronaviruses by Solar Radiation With Special Reference to COVID-19. Photochem Photobiol. (2020) 96:731–7. 10.1111/php.13293 32502327 PMC7300806

[8] Shi P Dong Y Yan H Zhao C Li X Liu W. Impact of temperature on the dynamics of the COVID-19 outbreak in China. Sci Total Environ. (2020) 728:138890. 10.1016/j.scitotenv.2020.138890 32339844 PMC7177086

[9] Adhikari A Yin J. Short-term effects of ambient ozone, PM(2.5,) and meteorological factors on COVID-19 confirmed cases and deaths in Queens, New York. Int J Environ Res Public Health. (2020) 17(11). 10.3390/ijerph17114047 PMC7312351 32517125

[10] Tosepu R Gunawan J Effendy DS Ahmad OAI Lestari H Bahar H. Correlation between weather and Covid-19 pandemic in Jakarta, Indonesia. Sci Total Environ. (2020) 725:138436. 10.1016/j.scitotenv.2020.138436 32298883 PMC7270847

[11] Sahin M. Impact of weather on COVID-19 pandemic in Turkey. Sci Total Environ. (2020) 728:138810. 10.1016/j.scitotenv.2020.138810 32334158 PMC7169889

[12] Bashir MF Ma B Bilal Komal B Bashir MA Tan D. Correlation between climate indicators and COVID-19 pandemic in New York, USA. Sci Total Environ. (2020) 728:138835. 10.1016/j.scitotenv.2020.138835 32334162 PMC7195034

[13] Zoran MA Savastru RS Savastru DM Tautan MN. Assessing the relationship between surface levels of PM2.5 and PM10 particulate matter impact on COVID-19 in Milan, Italy. Sci Total Environ. (2020) 738:139825. 10.1016/j.scitotenv.2020.139825 32512362 PMC7265857

[14] Qi H Xiao S Shi R Ward MP Chen Y Tu W. COVID-19 transmission in Mainland China is associated with temperature and humidity: a time-series analysis. Sci Total Environ. (2020) 728:138778. 10.1016/j.scitotenv.2020.138778 32335405 PMC7167225

[15] Malki Z Atlam ES Hassanien AE Dagnew G Elhosseini MA Gad I. Association between weather data and COVID-19 pandemic predicting mortality rate: machine learning approaches. Chaos Solitons Fractals. (2020) 138:110137. 10.1016/j.chaos.2020.110137 32834583 PMC7367008

[16] Sharma AK Balyan P. Air pollution and COVID-19: is the connect worth its weight? Indian J Public Health. (2020) 64:S132–4. 10.4103/ijph.IJPH_466_20 32496243

[17] Copat C Cristaldi A Fiore M Grasso A Zuccarello P Signorelli SS. The role of air pollution (PM and NO(2)) in COVID-19 spread and lethality: a systematic review. Environ Res. (2020) 191:110129. 10.1016/j.envres.2020.110129 32853663 PMC7444490

[18] Bukhari Q Massaro JM D'Agostino RB Sr. Khan S. Effects of weather on coronavirus pandemic. Int J Environ Res Public Health. (2020) 17:5399. 10.3390/ijerph17155399 PMC7432279 32727067

[19] Yao Y Pan J Liu Z Meng X Wang W Kan H. No association of COVID-19 transmission with temperature or UV radiation in Chinese cities. Eur Respir J. (2020) 55:2000517. 10.1183/13993003.00517-2020 32269084 PMC7144256

[20] Iqbal N Fareed Z Shahzad F He X Shahzad U Lina M. The nexus between COVID-19, temperature and exchange rate in Wuhan city: new findings from partial and multiple wavelet coherence. Sci Total Environ. (2020) 729:138916. 10.1016/j.scitotenv.2020.138916 32388129 PMC7194511

[21] Guo Y Gasparrini A Armstrong BG Tawatsupa B Tobias A Lavigne E. Heat Wave and mortality: a multicountry, multicommunity study. Environ Health Perspect. (2017) 125:087006. 10.1289/EHP1026 28886602 PMC5783630

[22] Gasparrini A Guo Y Hashizume M Lavigne E Zanobetti A Schwartz J. Mortality risk attributable to high and low ambient temperature: a multicountry observational study. Lancet. (2015) 386:369–75. 10.1016/S0140-6736(14)62114-0 26003380 PMC4521077

[23] Psistaki K Paschalidou AK McGregor G. Weather patterns and all-cause mortality in England, UK. Int J Biometeorol. (2020) 64:123–36. 10.1007/s00484-019-01803-0 31707494

[24] Fonseca-Rodríguez O Lundevaller EH Sheridan SC Schumann B. Association between weather types based on the spatial synoptic classification and all-cause mortality in Sweden, 1991–2014. Int J Environ Res Public Health. (2019) 16:1696. 10.3390/ijerph16101696 PMC6573000 31091805

[25] Ma Y Zhao Y Liu J He X Wang B Fu S. Effects of temperature variation and humidity on the death of COVID-19 in Wuhan, China. Sci Total Environ. (2020) 724:138226. 10.1016/j.scitotenv.2020.138226 32408453 PMC7142681

[26] Sajadi MM Habibzadeh P Vintzileos A Shokouhi S Miralles-Wilhelm F Amoroso A. Temperature, humidity, and latitude analysis to estimate potential spread and seasonality of coronavirus disease 2019 (COVID-19). JAMA Netw Open. (2020) 3:e2011834. 10.1001/jamanetworkopen.2020.11834 32525550 PMC7290414

[27] Cacho PM Hernández JL López-Hoyos M Martínez-Taboada VM. Can climatic factors explain the differences in COVID-19 incidence and severity across the Spanish regions?: an ecological study. Environ Health. (2020) 19:106. 10.1186/s12940-020-00660-4 33050915 PMC7552591

[28] Courtney JM Bax A. Hydrating the respiratory tract: an alternative explanation why masks lower severity of COVID-19 disease. medRxiv [Preprint]. (2020). 10.1101/2020.12.23.20248671 33582134 PMC7879047

[29] Sehra ST Salciccioli JD Wiebe DJ Fundin S Baker JF. Maximum daily temperature, precipitation, ultraviolet light, and rates of transmission of severe acute respiratory syndrome coronavirus 2 in the United States. Clin Infect Dis. (2020) 71:2482–7. 10.1093/cid/ciaa681 32472936 PMC7314246

[30] Wu Y Jing W Liu J Ma Q Yuan J Wang Y. Effects of temperature and humidity on the daily new cases and new deaths of COVID-19 in 166 countries. Sci Total Environ. (2020) 729:139051. 10.1016/j.scitotenv.2020.139051 32361460 PMC7187824

[31] Alam N Lindeboom W Begum D Streatfield PK. The association of weather and mortality in Bangladesh from 1983-2009. Glob Health Action. (2012) 5:53–60. 10.3402/gha.v5i0.19121 23195512 PMC3508913

[32] Luan GJ Yin P Wang LJ You JL Zhou MG. Association between high air temperature and mortality in summer: A multi-city analysis in China. Zhonghua Liu Xing Bing Xue Za Zhi. (2019) 40:59–63. 10.3760/cma.j.issn.0254-6450.2019.01.012 30669732

[33] Zhang K Li Y Schwartz JD O'Neill MS. What weather variables are important in predicting heat-related mortality? A new application of statistical learning methods. Environ Res. (2014) 132:350–9. 10.1016/j.envres.2014.04.004 24834832 PMC4091921

[34] Chen TH Li X Zhao J Zhang K. Impacts of cold weather on all-cause and cause-specific mortality in Texas, 1990-2011. Environ Pollut. (2017) 225:244–51. 10.1016/j.envpol.2017.03.022 28390302

[35] Lane K Ito K Johnson S Gibson EA Tang A Matte T. Burden and risk factors for cold-related illness and death in New York City. Int J Environ Res Public Health. (2018) 15:632. 10.3390/ijerph15040632 29601479 PMC5923674

[36] Nam YH Bilker WB Leonard CE Bell ML Alexander LM Hennessy S. Effect of statins on the association between high temperature and all-cause mortality in a socioeconomically disadvantaged population: a cohort study. Sci Rep. (2019) 9:4685. 10.1038/s41598-019-41109-0 30886182 PMC6423125

[37] Suwanwongse K Shabarek N. Hyperpyrexia in patients with COVID-19. J Med Virol. (2020) 92:2857–62. 10.1002/jmv.26154 32519768 PMC7300797

[38] de Bruijn R Romero LM. The role of glucocorticoids in the vertebrate response to weather. Gen Comp Endocrinol. (2018) 269:11–32. 10.1016/j.ygcen.2018.07.007 30012539

[39] Strong RA Silva EB Cheng HW Eicher SD. Acute brief heat stress in late gestation alters neonatal calf innate immune functions. J Dairy Sci. (2015) 98:7771–83. 10.3168/jds.2015-9591 26298746

[40] Jafari MJ Pirposhteh EA Dehghan SF Khodakarim S Jafari M. Relationship between heat stress exposure and some immunological parameters among foundry workers. Int J Biometeorol. (2020) 64:853–61. 10.1007/s00484-020-01874-4 32036432

[41] Niess AM Fehrenbach E Lehmann R Opavsky L Jesse M Northoff H. Impact of elevated ambient temperatures on the acute immune response to intensive endurance exercise. Eur J Appl Physiol. (2003) 89:344–51. 10.1007/s00421-003-0809-3 12736844

[42] Watkins ER Hayes M Watt P Renshaw D Richardson AJ. Extreme occupational heat exposure is associated with elevated haematological and inflammatory markers in Fire Service Instructors. Exp Physiol. (2020) 106:233–43. 10.1113/EP088386 32462715

[43] Tulapurkar ME Hasday JD Singh IS. Prolonged exposure to hyperthermic stress augments neutrophil recruitment to lung during the post-exposure recovery period. Int J Hyperthermia. (2011) 27:717–25. 10.3109/02656736.2011.601528 21992563

[44] Lecchi C Rota N Vitali A Ceciliani F Lacetera N. *In vitro* assessment of the effects of temperature on phagocytosis, reactive oxygen species production and apoptosis in bovine polymorphonuclear cells. Vet Immunol Immunopathol. (2016) 182:89–94. 10.1016/j.vetimm.2016.10.007 27863557

[45] Honda T Manjourides J Suh H. Daily ambient temperature is associated with biomarkers of kidney injury in older Americans. Environ Res. (2019) 179:108790. 10.1016/j.envres.2019.108790 31605868 PMC6893879

